# Exposed Pulps and Fang Filling

**Published:** 1870-03

**Authors:** Thos. J. Speck


### ARTICLE II.

### *Exposed Pulps and Fang Filling.*

By THOS. J. SPECK, D. D. S.

The writer of this paper proposes to review the various methods of treating exposed pulps with a view to the preservation of their vitality, together with the treatment of fangs or roots of the teeth after the removal of the nerve, and the most approved method of "fang filling."

Some operators, whose position in the profession is such that their opinions and practice are at least entitled to attention, claim that after the pulp has become exposed, inflammation of the periosteum set up and even suppuration of a portion of the pulp commenced, that by excising the most diseased portion, and using certain therapeutical treatment, such as the application of astringents, escharotics and anodynes, followed by filling with some non-conducting material, that the diseased pulp can be restored to a healthy and normal state. The propriety of such practice is questionable. It is useless to speak of the highly sensitive and delicate nature of the dental pulp, this is presumed to be understood. The writer of this has attempted the practice in several most favorable cases, all of which resulted in complete failure. Others claim that by capping with gold, lead, tin, horn and oiled silk, that the vitality of the pulp may be preserved. At one time it was believed practicable, and many thought their operations a success; some patients complaining of slight dull pain—not of sufficient importance to apply for relief; others that no pain followed whatever. And what dental practitioner has not met with many cases of persons losing their entire complement of teeth, without suffering the slightest pain—known as sensitive or toothache? Probably the painless cases belong to such a diathesis. The sequel of all such operations proved entire failures, and the practice has been abandoned by most operators. On examination of the most favorable cases after one, two, or three years, the pulp was found entirely dead—some of the teeth producing so much pain and trouble, as to necessitate their removal; others remaining comparatively quiet, though dead.

In treating exposed pulps the only successful cases are those having no serious inflammation, and where this organ has been accidentally exposed by unskillful excavation; for there is no reason why the pulp of a tooth, in certain cases, may not be restored to health as well as other parts of the system, having nutrition, circulation and absorption. Several such instances of filling with gold over a slightly exposed pulp, without inflammation or wounding the membrane, ten years ago, and the teeth still retaining their vitality and color, can be referred to in the practice of the writer. The practice of filling over diseased pulps, as claimed by some good operators, belongs to the past—has been found a waste of time, impracticable and uncertain. It is very nice to tell about, but to practice successfully is another thing. But some men, in fact all, have their "hobbies"—let them ride them in talk—but the conscientious dentist is always careful in making positive promises to his patients, that he knows to be doubtful even at best. Unfortunately for us as operators, our patients never apply for treatment of diseased pulps until all the parts are involved, so much so that restoration, if at all possible at a more favorable time, is highly improbable. What then, in justice to our patients is our duty? To give them advice and work that will prove truthful and beneficial, and will not bring self accusation on ourselves. Then we can maintain a position independent and secure the confidence and esteem all earnest laborers deserve. There is a mode by which such teeth can be preserved almost painlessly, and in many cases be made as useful as ever, and in appearance and service equal to living teeth. This plan is the extirpation of the pulp—reducing the parts to a healthy condition, and thoroughly filling both nerve canal and pulp cavity. The various methods of securing this result, depends to a considerable extent upon the condition of the mouth and tooth and the age of the patient. For a successful operation it is necessary to restore the adjacent parts to a normal state, as well as the tooth. In recently exposed pulps the best practice, according to the writer's experience, is to extirpate the pulp by the surgical or "Heroic" operation. Armed with a well tempered barbed broach—and much depends on a good instrument—we extract the pulp directly, using some one of the various anæsthetic agents—the ether spray being preferred; and in cases of good strong constitution, as soon as the hæmorrhage ceases the tooth may be filled. Great care should be taken to remove all debris, which is often very difficult. Failures result sometimes on account of slovenly practice in this respect. Small particles of dentine—almost an impalpable powder—may be forced through the orifice of the canal of the fang, and being foreign, is a source of irritation. Now, the cavity and nerve canal being prepared, and the tooth ready for the filling, what shall we use? There are various materials and each has its warm advocates. It is potent to all that the material which can be used with greatest facility and condensed most thoroughly is best. Some advocate shaping a piece of seasoned hickory wood, and before inserting saturate with creasote. The question arises, Will not wood, as it is very porous, absorb the fluids and gases thrown off by the excised portion of the nerve and inflamed periosteum and thus produce disease? And the wood is liable to be forced through the canal at the apex of the fang, and produce irritation. Others advocate filling a portion of the fang with cotton saturated with carbolic acid or creasote. Others again, to fill the fang entire with os artificial, or some of the various plastic fillings, which practice is of doubtful propriety. In fact it seems almost impossible to thoroughly fill so long and narrow a cavity with such material, and half filled is as poor as no filling at all. How can such material be carried through the windings of some nerve canals, and how can we be satisfied the filling is thorough and entire? The nature of the material is such that the orifice would become choked, and the operator be deceived as to the completeness of the filling. The opinion and experience of the writer is that the best material is No 2 or 4 plain gold foil. The best manner to introduce the first gold is to roll a few folds of foil around a very fine smooth broach and carefully carry as far up the canal as possible, and then thoroughly consolidate. By exercising care in forming the roll there is no danger of forcing the gold through the foramen at the apex of the fang. The next gold may be introduced in the same way, or in ribbons or ropes as the operator prefers or finds most convenient, so that every portion of the canal is firmly and densely filled. Then proceed to fill the crown as in other cavities.

When the direct extirpation of the living pulp is impracticable, it must be devitalized before extraction. The devitalizing agent generally employed at the present day is arsenious acid, and this is considered the most certain and least injurious to the tooth structure. Still all must admit that an agent so powerful, and that acts so rapidly and surely on the nerve substance, leaves some of its destructive properties in the surrounding parts. A general discussion of its influences will not now. be made Its action is strange and unaccountable. In some instances it may be applied to an exposed pulp in an inflamed condition, the tooth giving the most excruciating and acute pain, and the application render immediate relief. At other times in the same mouth and under the same pathological conditions, it will increase the pain. Some writers contend that when it is applied to an exposed and lacerated pulp, pain will follow; while others assert as confidently, that when applied to a pulp with a slight wall of dead dentine intervening, that pain will ensue; all of which in the practice of the writer has been found true, consequently no positive theory has as yet been established. Its freaks are peculiar and no hypothesis has yet been advanced to account for the various effects. It cannot be said—with due respect to the opinion of others—that the various effects depend on the peculiar diathesis or characteristics of the patient, for this assertion is made from actual experiment; that arsenious acid has been applied in two teeth pathologically the same in every respect—even adjoining teeth, and different results followed. However, with all its shines, it is a kindly agent, both to operator and patient, in a great many instances. There are many difficult and almost inaccessible pulp cavities which we are called upon to treat, and without the aid of some ready and certain devitalizing agent our efforts would be vain. Such as highly sensitive and nervous temperaments—teeth with tortuous nerve canals, and when the operation of extracting the pulp would require time and care, it is essential. The manner of application is known to all, and to repeat it here is unnecessary. Nevertheless, great care and caution should be exercised in its use. In most cases one application, properly applied is sufficient, and the length of time to remain in the tooth is from 12 to 24 hours. It is well then to remove all extraneous or loose matter and carefully syringe with tepid water, and then seal the cavity with cotton and sandarach varnish, and after the lapse of four or five days the pnlp may be removed comparatively without pain. The next step then is to carefully prepare and shape the crown and pulp cavity and dress with creasote or carbolic acid, and dismiss the patient for another day. If no inflammation or signs of periostitis supervene the tooth may be filled with impunity, but should suppuration or discharge of fetid matter occur, which can readily be ascertained by introducing floss silk or wisps of cotton, the tooth should be dressed daily with iodine, creasote or carbolic acid, until all discharge ceases. Some operators prefer to insert a test filling of silk, to remain for a few days, which may be a good precautionary measure, but in many cases we cannot demand so much time of our patients, and when a practitioner educates his eye to minutely diagnose the parts around a tooth as described, he will not have many failures. Should abscess follow, the writer has found it best, if he has access to the case before an opening has been made for the discharge of pus through the alveolar ridge, to treat externally—that is if the filling is a good one let it remain—with a small, slightly curved hatchet shaped excavator, cut freely down through the alveolus to the apex of the fang, and tear up the forming sac, and insert a seaton thoroughly saturated with iodine and chloroform, creasote or carbolic acid, and dress once or twice a day as convenient. When properly conducted and under ordinary circumstances, a permanent cure may be expected.

There is another class of pulp cavities, the treatment of which give us more trouble and require a greater amount of skill and patience than the foregoing, which are teeth whose pulps are already dead. Great care should be taken in preparing the entire mouth, by removing all pre-existing causes of irritation, and as much as possible get the mouth in a healthy condition, before any action has been taken with the tooth. First, I remove all loose dead matter, and dress with agents well known to all operators, before excavating or shaping the cavity. Such cases require more energetic therapeutical treatment than any class of diseased teeth. After all evidence of inflammation has disappeared, and all acrid or offensive matter has been removed, the cavity may be prepared by gentle excavation, and letting it rest a few days, using at the same time the dressings named in the preceeding cases. Now if a test filling is of any advantage it is best employed in cases of this character. The fang should be filled with floss silk, and the crown with some solid temporary filling. After a reasonable time— say from three to ten days, and the tooth remains perfectly quiet, a permanent filling may be inserted as in the other classes, and in a majority of cases with entire success. If the profession would exercise more patience and skill, and teach their patients the great value of even dead teeth correctly treated, many valuable organs that are daily sacrificed might be preserved, and at the same time build lasting monuments to our skill and progressive science.

---

